# Clinical outcomes of cases requiring touch-up applications in pulmonary vein isolation with balloon ablation

**DOI:** 10.1016/j.hroo.2025.01.009

**Published:** 2025-01-28

**Authors:** Koshiro Kanaoka, Koji Miyamoto, Yoshitaka Iwanaga, Michikazu Nakai, Reina Tonegawa-Kuji, Yoko Sumita, Koichi Inoue, Teiichi Yamane, Akihiko Nogami, Yoshihiro Miyamoto, Wataru Shimizu, Kengo Kusano

**Affiliations:** 1 Department of Medical and Health Information Management, National Cerebral and Cardiovascular Center, Osaka, Japan; 2 Department of Cardiovascular Medicine, Nara Medical University, Kashihara, Japan; 3 Department of Cardiovascular Medicine, National Cerebral and Cardiovascular Center, Osaka, Japan; 4 Cardiovascular Division, National Hospital Organization Osaka National Hospital, Osaka, Japan; 5 Division of Cardiology, Department of Internal Medicine, Jikei University School, Minato City, Japan; 6 Department of Cardiology, Institute of Medicine, University of Tsukuba, Tsukuba, Japan; 7 Department of Cardiovascular Medicine, Nippon Medical School, Tokyo, Japan

**Keywords:** Touch-up application, Pulmonary vein isolation, Atrial fibrillation, Balloon ablation

## Abstract

**Background:**

Balloon ablation for pulmonary vein isolation (PVI) is a well-established treatment option for atrial fibrillation. Although some patients require touch-up ablation, generalizable evidence is limited.

**Objective:**

This study aimed to investigate the current status and outcomes of touch-up applications using a nationwide registry in Japan.

**Methods:**

Patients ≥18 years of age who underwent first-time PVI between January 2017 and December 2020 were included using the data from the Japanese Catheter Ablation registry. The annual trends in the proportion of cases requiring touch-up ablation with radiofrequency ablation were determined, and the associations of ablation strategies with acute success and periprocedural complications were analyzed using logistic regression analysis.

**Results:**

Of the 51,402 patients included, 28,412 and 22,990 patients underwent PVI using radiofrequency ablation and balloon ablation, respectively. In the balloon ablation group, 1462 (6.4%) patients required touch-up applications, and the proportion of cases requiring touch-up applications decreased during the study period from 9.5% in 2017 to 5.5% in 2020 (*P* for trend < .001). The proportion of acute success was >99% across all ablation strategies. Although 2.5% of the patients in the touch-up ablation group had phrenic nerve palsy, the composite of complications, except for phrenic nerve palsy, was not significantly increased in the balloon + touch-up ablation group compared with that in the balloon ablation–only group and radiofrequency ablation group.

**Conclusion:**

Touch-up applications following balloon ablation are required in some cases. Touch-up ablation with radiofrequency ablation may be a treatment option when achieving successful PVI using balloon ablation is difficult.

**Key Findings**

▪ A nationwide registry on catheter ablation in Japan revealed that 6.4% of patients underwent touch-up applications after balloon ablation, and the proportion decreased year by year.
▪ Patients requiring touch-up applications had complicated baseline characteristics.
▪ The proportion of acute success and periprocedural complications, except for phrenic nerve palsy, were similar between patients in the balloon + touch-up ablation group and those in the balloon ablation–only and radiofrequency ablation groups.

## Introduction

Catheter ablation is an established and effective treatment strategy for atrial fibrillation (AF).^1^ Compared with medical therapy, catheter ablation reduces rehospitalization in patients with heart failure and increases the maintenance of sinus rhythm.^2^^,^^3^ Catheter ablation of AF is primarily performed with pulmonary vein isolation (PVI).^4^ Balloon-based catheter ablations have emerged as an alternative strategy to conventional radiofrequency (RF) catheter ablation for achieving PVI.^5^, ^6^, ^7^ Balloon ablation has shown similar clinical efficacy to RF ablation, and prior studies have demonstrated that balloon ablation is associated with a shorter procedure duration and a lower complication rate.^8^, ^9^, ^10^, ^11^

Despite the widespread use of balloon technology, treatment failure of PVI can occur owing to anatomical and electrophysiologic factors. Although the device is designed to isolate PV with a single shot, optimal PV occlusion is sometimes hard to achieve.^12^ To complete PVI, touch-up applications using RF technology at the site of residual PV conduction are needed in some cases following balloon ablation. However, there is a lack of studies comprehensively evaluating the current situation and the safety of touch-up applications with RF ablation after balloon ablation.

Consequently, we aimed to investigate the current practices regarding touch-up applications with RF ablation after balloon ablation, as well as the efficacy and safety of touch-up applications, using a nationwide registry for catheter ablation in Japan.

## Methods

### Study design and data source

This study analyzed data from the Japanese Catheter Ablation (J-AB) registry, a nationwide prospective registry managed by the Japanese Heart Rhythm Society (JHRS) using an electronic data capture system.^13^^,^^14^ This study is registered at the UMIN Clinical Trial Registry (UMIN000028288) and ClinicalTrials.gov (NCT03729232). The AF ablation procedures using balloon ablation are available only at JHRS-certified facilities, and participation in the J-AB registry is mandatory to be a JHRS-certified arrhythmia training facility. A total of 480 facilities participated in this registry until December 2020, and the number of registered cases accounts for ≥90% of all catheter ablation procedures performed in Japan. The registry includes patients who underwent catheter ablation for any arrhythmia and collects data on patient characteristics, ablation procedures, and outcomes including acute success and complications. The J-AB registry collects detailed patient characteristics, procedures, and 1-year outcomes during its annual survey every September (September survey). The operating physician or research staff performs the data registration during or soon after the index hospitalization. The baseline survey form is completed in almost all cases.

### Patients

**Figure 1:**
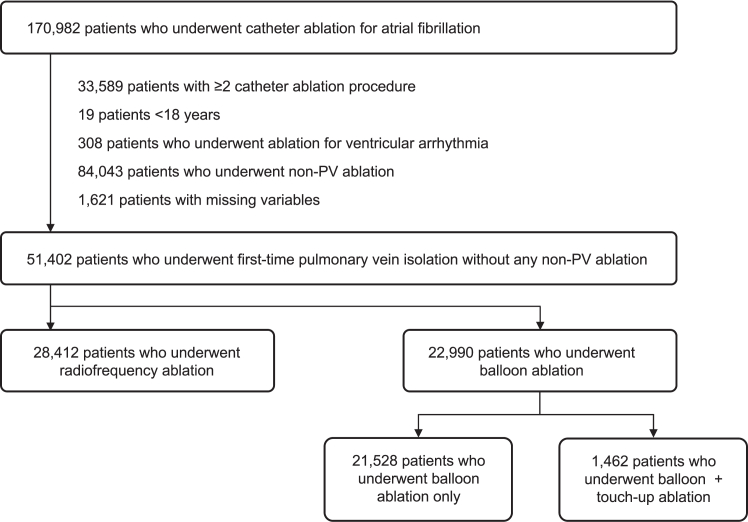
Flowchart of patient selection. A total of 51,413 patients who underwent first-time pulmonary vein isolation were included. Among them, 28,419 patients underwent radiofrequency ablation, and 22,994 patients underwent balloon ablation. PV = pulmonary vein.

Patients ≥18 years of age who underwent their first catheter ablation for AF between January 2017 and December 2020 were included. To investigate the efficacy and safety of PVI at the initial catheter ablation session, we excluded the following patients: (1) patients who underwent concomitant catheter ablation for ventricular arrhythmia; (2) patients who underwent non-PV ablation; and (3) patients with missing variables (Figure 1). First, the included patients were divided into 2 groups according to the ablation strategy: the RF ablation group and the balloon ablation group. Cryoballoon, hot balloon, and laser balloon were the approved balloon ablation technologies in Japan during the study period. Second, we described the annual trend of the proportion of cases requiring touch-up RF ablation at sites of residual or dormant PV conduction. Finally, patients in the balloon ablation group were divided into 2 groups according to whether or not they required touch-up applications using RF catheter: the balloon-only group and the balloon + touch-up ablation group. A cryocatheter can be used following cryoballoon ablation, but it was rarely used PVI strategy alone among patients in this database. We compared patient characteristics and outcomes in the touch-up ablation group with those in the balloon-only group and the RF ablation group.

### Outcome measures

The primary outcome measure was acute success and a composite measure of periprocedural complications following PVI. Acute success was defined as the achievement of bidirectional conduction block of the PVs. Periprocedural complications included cardiac tamponade, bleeding, phrenic nerve palsy, embolic events, acute gastric atony, pericarditis, bradycardia, pneumothorax, and acute heart failure associated with PVI. The secondary outcome measure was each periprocedural complication and the recurrence of atrial tachyarrhythmia (AF, atrial flutter, or atrial tachycardia) at 1 year. Data for the recurrence of atrial tachyarrhythmia were extracted from the annual September survey.

### Statistical analysis

Patient characteristics are described as number and percentage for categorical variables and as median (interquartile range) for continuous variables. The trends in the proportion of cases requiring touch-up applications were evaluated using the Cochran-Armitage test. The association between patient baseline characteristics and ablation technology or touch-up applications was analyzed using the chi-square test or Mann-Whitney *U* test. Outcome measures were compared among the RF ablation group, balloon ablation–only group, and balloon + touch-up ablation group. Univariable and multivariable logistic regression analyses were conducted to determine the association between the groups and primary outcomes. The variables included in the model were age, sex, body mass index, type of AF, symptoms, failed antiarrhythmic drugs, history of stroke or embolic event, and comorbidities. Kaplan-Meier curves and Cox proportional hazards models were used to analyze the association between the groups and recurrence of atrial tachyarrhythmia. Statistical significance was set at *P* < .05. Bonferroni correction was used when a post hoc analysis for outcomes was performed. All statistical analyses were performed using Stata version 16 (StataCorp).

### Ethics statements

This study was approved by the ethics committee of the National Cerebral and Cardiovascular Center (registration number: M28-114-11) and was conducted in accordance with the principles of the Declaration of Helsinki. All participants provided written informed consent or could opt out and could withdraw their consent at any time.

## Results

### Patient characteristics according to the ablation technology

Of the 170,982 patients who underwent catheter ablation for AF, 51,402 patients who underwent first-time PVI without any non-PV ablation were included (Figure 1). Among them, 28,412 (55.3%) patients underwent RF ablation and 22,990 (44.7%) patients underwent balloon ablation. The proportion of patients with paroxysmal AF and those with symptomatic AF was higher in the balloon ablation group than in RF ablation group (Table 1). Patients in the RF ablation group had more cardiovascular comorbidities such as cardiomyopathy and valvular disease.

**Table 1:** Patient characteristics according to the ablation strategy

	RF (n = 28,412)	Balloon (n = 22,990)	*P* value
Age, y	69 (60–75)	68 (60–75)	.002
Female	8824 (31.1)	7692 (33.5)	<.001
Body mass index, kg/m^2^	24.1 (21.9–26.6)	23.7 (21.7–26.2)	<.001
Type of atrial fibrillation			<.001
Paroxysmal	16,339 (57.5)	19,828 (86.2)	
Persistent or long-standing	12,083 (42.5)	3162 (13.8)	
Symptom			<.001
No	4771 (16.8)	2638 (11.5)	
Yes	22,982 (80.9)	19,855 (86.3)	
Unknown	659 (2.3)	497 (2.2)	
Failed antiarrhythmic drugs	10,974 (38.6)	8489 (36.9)	<.001
Comorbidities			
Ischemic heart disease	1814 (6.4)	1399 (6.1)	.16
Cardiomyopathy	1637 (5.8)	657 (2.9)	<.001
Valvular disease	648 (2.3)	273 (1.2)	<.001
History of stroke/embolic event			<.001
No	25,816 (90.9)	21,157 (92.0)	
Yes	1929 (6.8)	1333 (5.8)	
Unknown	667 (2.3)	500 (2.2)	
Balloon technologies			<.001
Cryoballoon	—	20,478 (89.1)	
Hot balloon	—	1392 (6.1)	
Laser balloon	—	1120 (4.9)	
Year of the index procedure			
2017	1215 (4.3)	960 (4.2)	<.001
2018	6205 (21.8)	5832 (25.4)	
2019	10,198 (35.9)	8427 (36.7)	
2020	10,794 (38.0)	7771 (33.8)	
Touch-up	—	1462 (6.4)	

Values are or median (interquartile range) or n (%).

RF = radiofrequency ablation.

### Patient characteristics according to touch-up applications

**Figure 2:**
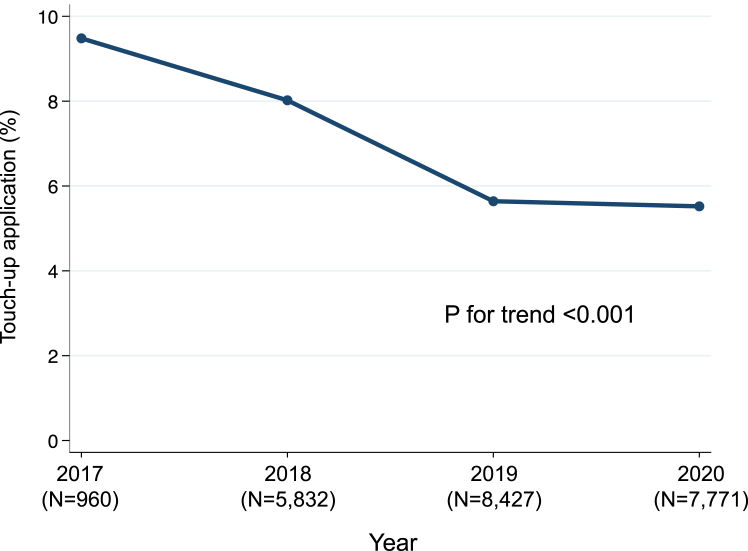
Trends in the proportion of cases requiring touch-up radiofrequency ablation after balloon ablation. The proportion decreased from 9.5% in 2017 to 5.5% in 2020.

In the balloon ablation group, 1462 (6.4%) patients underwent touch-up applications. The proportion of cases requiring touch-up applications decreased during the study period from 9.5% in 2017 to 8.0% in 2018, 5.6% in 2019, and 5.5% in 2020 (*P* for trend < .001) (Figure 2). Patients who underwent touch-up applications were more likely to have persistent AF, a history of drug-resistant AF, and cardiovascular comorbidities than were patients who did not (Table 2). The proportion of cases requiring touch-up applications was higher in patients who underwent hot balloon ablation (19.8% [n = 275 of 1392]) than in those who underwent cryoballoon ablation (5.5% [n = 1121 of 20,478]) or laser balloon ablation (5.9% [n = 66 of 1120]).

**Table 2:** Patient characteristics according to touch-up applications

	Balloon ablation only (n = 21,528)	Balloon + touch-up ablation (n = 1462)	*P* value
Age, y	68 (60–75)	69 (60–75)	.36
Female	7206 (33.5)	486 (33.2)	.86
Body mass index, kg/m^2^	23.7 (21.7–26.1)	23.9 (21.7–26.4)	.09
Type of atrial fibrillation			<.001
Paroxysmal	18,657 (86.7)	1171 (80.1)	
Persistent or long-standing	2871 (13.3)	291 (19.9)	
Symptom			.083
No	2444 (11.4)	194 (13.3)	
Yes	18,617 (86.5)	1238 (84.7)	
Unknown	467 (2.2)	30 (2.1)	
Failed antiarrhythmic drugs	7873 (36.6)	616 (42.1)	<.001
Comorbidities			
Ischemic heart disease	1289 (6.0)	110 (7.5)	.017
Cardiomyopathy	603 (2.8)	54 (3.7)	.047
Valvular disease	238 (1.1)	35 (2.4)	<.001
History of stroke/embolic event			.79
No	19,815 (92.0)	1342 (91.8)	
Yes	1243 (5.8)	90 (6.2)	
Unknown	470 (2.2)	30 (2.1)	
Balloon technologies			<.001
Cryoballoon	19,357 (89.9)	1121 (76.7)	
Hot balloon	1117 (5.2)	275 (18.8)	
Laser balloon	1054 (4.9)	66 (4.5)	

Values are median (interquartile range) or n (%).

RF = radiofrequency ablation.

### Acute success and periprocedural complications according to the ablation strategy

There was a high proportion of acute success across all ablation strategies (99.1% in the RF ablation group, 99.4% in the balloon ablation–only group, and 99.5% in the balloon + touch-up ablation group) (Table 3). The proportion of patients with any complications was higher in the balloon + touch-up ablation group (4.2%) than in the RF ablation group (2.3%) and the balloon ablation–only group (2.4%) (*P* < .001). In the balloon + touch-up ablation group, 2.5% of the patients had phrenic nerve palsy. The proportions of any complication except for phrenic nerve palsy and cardiac tamponade were higher in the RF ablation group (2.2% and 1.0%, respectively) than in the balloon ablation–only group (1.6% and 0.5%, respectively) (*P* < .001). There was no significant difference in any complications, except for phrenic nerve palsy, between the balloon ablation–only group and the balloon + touch-up ablation group (*P* = .56). Multivariable logistic regression analysis showed that acute complications, except for phrenic nerve palsy, were higher in the RF ablation–only group than in the balloon ablation–only group (odds ratio [OR] 1.37, 95% confidence interval [CI] 1.20–1.58, *P* < .001), and these complications were not significantly increased in the balloon + touch-up ablation group compared with those in the balloon ablation–only group (OR 1.10, 95% CI 0.73–1.64, *P* = .66) and the RF ablation group (OR 0.80, 95% CI 0.54–1.19, *P* = .27) (Table 4).

**Table 3:** Acute success and periprocedural complications according to ablation strategies

	Radiofrequency ablation (n = 28,412)	Balloon ablation only (n = 21,528)	Balloon ablation + touch-up (n = 1462)	*P* value
Acute success	28,152 (99.1)	21,407 (99.4)	1455 (99.5)	<.001
Periprocedural complications				
Composite of any complication	643 (2.3)	525 (2.4)	62 (4.2)	<.001∗^,^†
Composite of any complication, except for phrenic nerve palsy	633 (2.2)	340 (1.6)	26 (1.8)	<.001
Cardiac tamponade	291 (1.0)	102 (0.5)	13 (0.9)	<.001†
Hematoma	112 (0.4)	95 (0.4)	5 (0.3)	.66
Other bleedings	25 (0.1)	20 (0.1)	1 (0.1)	.95
Phrenic nerve palsy	10 (0.0)	193 (0.9)	37 (2.5)	<.001∗^,^†
Stroke or embolic events	61 (0.2)	56 (0.3)	4 (0.3)	.56
Acute gastric atony	46 (0.2)	19 (0.1)	1 (0.1)	.061
Pericarditis	39 (0.1)	20 (0.1)	0 (0.0)	.15
Sick sinus syndrome	34 (0.1)	13 (0.1)	1 (0.1)	.095
Atrioventricular block	3 (0.0)	4 (0.0)	0 (0.0)	.68
Pneumothorax	8 (0.0)	11 (0.1)	0 (0.0)	.32
Heart failure	24 (0.1)	7 (0.0)	1 (0.1)	.07

∗ Significantly different between the radiofrequency ablation group and the balloon + touch-up ablation group on post hoc analysis.

† Significantly different between the balloon ablation–only group and balloon + touch-up ablation group on post hoc analysis.

**Table 4:** Complications except for phrenic nerve palsy by ablation strategy

	Univariable analysis		Multivariable analysis	
	OR (95% CI)	*P* value	OR (95% CI)	*P* value
Radiofrequency ablation	1.42 (1.23–1.62)	<.001	1.37 (1.20–1.58)	<.001
Balloon ablation	Reference		Reference	
Balloon ablation + touch-up	1.13 (0.75–1.69)	.56	1.10 (0.73–1.64)	.66

Multivariable analysis was adjusted with age, sex, body mass index, type of atrial fibrillation, atrial fibrillation symptoms, failed antiarrhythmic drugs, history of stroke or embolic event, and comorbidities. The OR in the balloon + touch-up ablation group, with the radiofrequency ablation group as reference, was 0.80 (95% CI 0.54–1.19, *P* = .27).

CI = confidence interval; OR = odds ratio.

### Comparison of 1-year recurrence between the groups

**Figure 3:**
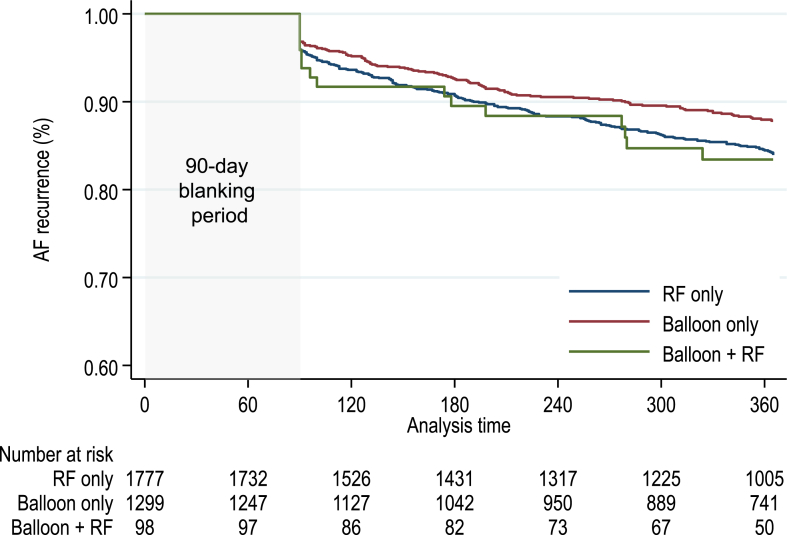
Kaplan-Meier curve of freedom from atrial fibrillation (AF) recurrence. There were no significant differences between groups. RF = radiofrequency.

Of 3690 patients registered in the September survey, 3174 with follow-up data and without missing variables were analyzed. The patient characteristics obtained from the annual September survey are shown in Supplemental Table 1. Patients in the RF ablation group had higher B-type natriuretic peptide and N-terminal pro–B-type natriuretic peptide levels, and the EnSite mapping system or no mapping system was used more in patients in the balloon + touch-up ablation group. The median left atrial diameter was 39 mm (interquartile range 34–42 mm) in the balloon ablation group and 40 mm (interquartile range 35–44 mm) in the balloon + touch-up group. The procedure time was longer in the RF ablation group than in the balloon ablation–only group and the balloon + touch-up ablation group. Univariable and multivariable Cox proportional hazards models showed no significant difference in the recurrence among the groups (Figure 3; Supplemental Table 2).

## Discussion

Generalizable evidence for touch-up ablation after balloon ablation for PVI is inadequate. The current study found the following substantial results. First, only a small proportion of patients (6.4%) underwent touch-up applications, and this proportion decreased year by year. Second, patients requiring touch-up applications had complicated background characteristics, and a higher proportion of them underwent hot balloon ablation. Third, acute success and periprocedural complications, except for phrenic nerve palsy, were similar between patients in the balloon + touch-up ablation group and those in the balloon ablation–only group and the RF ablation group. To the best of our knowledge, this is the largest study to date to investigate touch-up applications following balloon ablation. Although we need to be careful about the occurrence of phrenic nerve palsy, touch-up applications could be a reasonable option when it is difficult to ensure the success of PVI using balloon ablation.

### Current status of touch-up applications

This study showed that <10% of patients required touch-up applications after balloon ablation. A recent study in Germany reported a 97% acute success rate.^15^ In a study by Wei and colleagues,^16^ approximately 4.0% of patients required touch-up applications. These results indicate that it is difficult to achieve success in some PVI procedures even in the current clinical setting. However, we showed that the proportion of patients requiring touch-up applications has decreased. Balloon ablation is characterized by a short learning curve, and touch-up applications may be less necessary in the future.^17^ Although our study period did not include the data on pulsed field ablation, the increase in the case of pulsed field ablation may lead to a reduction of touch-up applications among patients who underwent one-shot device.

Our study also showed that patients with persistent AF and cardiovascular comorbidities were more likely to require touch-up applications. A prior study showed that patients with heart failure have a higher probability of requiring touch-up applications.^18^ Another study revealed that anatomical variations were associated with the need for multiple applications, and it may be difficult to achieve PVI in patients with complex conditions.^19^ The current study also found that using hot balloon ablation for PVI was associated with a higher risk of requiring touch-up applications. This is consistent with previous findings that touch-up applications frequently occur after hot balloon ablation.^12^^,^^20^

Although our study did not investigate detailed anatomical factors predictive of touch-up applications, baseline characteristics such as cardiovascular comorbidities and balloon strategies may be useful in predicting the need for touch-up applications.

### Efficacy and safety of balloon ablation as a first-line PVI technique

In the current study, the rates of acute success and 1-year recurrence were similar between the RF ablation group and the balloon ablation–only group. Among all complications, phrenic nerve palsy occurred more frequently in the balloon + touch-up ablation group; this may be because the patients in this group changed their ablation strategy to touch-up ablation after the onset of phrenic nerve palsy. The proportion of complications, except for phrenic nerve palsy, was higher in the RF ablation group than in the balloon ablation–only group, especially cardiac tamponade. The higher proportion of complications in the RF ablation group was consistent with prior findings.^8^^,^^21^ Balloon ablation is a safe option for achieving PVI and has a shorter procedure time. Thus, first-line PVI using balloon ablation is a reasonable treatment option. Although we should be careful about the occurrence of phrenic nerve palsy, touch-up using RF ablation can lead to complete PVI when PVI is not successful by balloon ablation.

### Limitations

Although our study used data from a nationwide real-world cohort, it still has several limitations. First, there may be unmeasured confounders associated with touch-up applications, such as anatomical, left atrial volume, and electrophysiologic factors, and blood test results. Second, we do not have detailed data on the number of PVIs performed using balloon ablation. Therefore, a large study using detailed data on these characteristics is warranted. Finally, the data from the registry do not include detailed information on the recovery after complications such as phrenic nerve palsy.

## Conclusion

Only a small proportion of patients who undergo PVI using balloon ablation require touch-up applications, and the proportion has decreased over time. Importantly, if we take care of the occurrence of phrenic nerve palsy, touch-up applications with RF ablation could be a reasonable option when it is difficult to achieve success with PVI using balloon ablation.
