# Supplementary material for: Clinical outcomes of cases requiring touch-up applications in pulmonary vein isolation with balloon ablation
Source: Heart Rhythm O2. 2025 Jan 28;6(4):410–6. doi: 10.1016/j.hroo.2025.01.009 (PMC12047475; doi:10.1016/j.hroo.2025.01.009)
Supplement: Supplemental Tables 1 and 2 [file mmc1.docx]

**SUPPLEMENTAL APPENDIX**

**Clinical Outcomes of Cases Requiring Touch-up Applications in Pulmonary Vein Isolation With Balloon Ablation**

Koshiro Kanaoka, MD, PhD; Koji Miyamoto MD, PhD; Yoshitaka Iwanaga, MD, PhD; Michikazu Nakai, PhD; Reina Tonegawa-Kuji MD, PhD; Yoko Sumita; Koichi Inoue MD, PhD; Teiichi Yamane; Kengo Kusano, MD, PhD

**Corresponding author:** Kengo Kusano

Department of Cardiovascular Medicine, National Cerebral and Cardiovascular Center, Kishibe-Shimmachi 6-1, Suita, Osaka 564-8565, Japan

E-mail: [kusanokengo@ncvc.go.jp](mailto:kusanokengo@ncvc.go.jp)

**Supplemental Appendix:**

Page 1. Title page

Pages 2-5. Supplemental Table 1
Page 6. Supplemental Table 2

| Supplemental Table 1. Patient characteristics according to ablation strategies | | | | | | | | | |
| --- | --- | --- | --- | --- | --- | --- | --- | --- | --- |
|  | Radiofrequency ablation | |  | Balloon ablation | |  | Balloon + touch-up | | P-value |
|  | Available N |  |  | Available N |  |  | Available N |  |  |
| Age, years | 1,777 | 69 (61–75) |  | 1,299 | 69 (61–75) |  | 98 | 68 (59–74) | 0.63 |
| Female sex | 1,777 | 599 (33.7) |  | 1,299 | 435 (33.5) |  | 98 | 32 (32.7) | 0.97 |
| Body mass index, kg/m^2^ | 1,777 | 24.0 (21.9–26.4) |  | 1,299 | 23.6 (21.6–25.9) |  | 98 | 23.7 (20.9–25.6) | 0.006 |
| Type of AF | 1,777 |  |  | 1,299 |  |  | 98 |  |  |
| Paroxysmal |  | 1,047 (58.9) |  |  | 1,127 (86.8) |  |  | 80 (81.6) | <0.001 |
| Persistent |  | 730 (41.1) |  |  | 172 (13.2) |  |  | 18 (18.4) |  |
| Symptom of AF | 1,777 |  |  | 1,299 |  |  | 98 |  |  |
| Unsymptomatic |  | 201 (11.3) |  |  | 95 (7.3) |  |  | 5 (5.1) | <0.001 |
| Symptomatic |  | 957 (53.9) |  |  | 774 (59.6) |  |  | 63 (64.3) |  |
| Unknown |  | 619 (34.8) |  |  | 430 (33.1) |  |  | 30 (30.6) |  |
| Failed AADs | 1,777 | 685 (38.5) |  | 1,299 | 476 (36.6) |  | 98 | 35 (35.7) | 0.52 |
| No. of previous AADs | 1,777 |  |  | 1,299 |  |  | 98 |  | 0.96 |
| None |  | 1,092 (61.6) |  |  | 823 (63.4) |  |  | 63 (64.3) |  |
| 1 |  | 515 (29.0) |  |  | 365 (28.1) |  |  | 26 (26.5) |  |
| 2 |  | 144 (8.1) |  |  | 95 (7.3) |  |  | 7 (7.1) |  |
| ≥3 |  | 22 (1.2) |  |  | 16 (1.2) |  |  | 2 (2.0) |  |
| Comorbidities |  |  |  |  |  |  |  |  |  |
| Hypertension | 1,777 | 802 (45.1) |  | 1,299 | 571 (44.0) |  | 98 | 43 (43.9) | 0.80 |
| Diabetes | 1,777 | 249 (14.0) |  | 1,299 | 172 (13.2) |  | 98 | 11 (11.2) | 0.65 |
| Dyslipidemia | 1,777 | 391 (22.0) |  | 1,299 | 308 (23.7) |  | 98 | 21 (21.4) | 0.51 |
| IHD | 1,777 | 157 (8.8) |  | 1,299 | 118 (9.1) |  | 98 | 5 (5.1) | 0.41 |
| Cardiomyopathy | 1,777 | 111 (6.2) |  | 1,299 | 31 (2.4) |  | 98 | 2 (2.0) | <0.001 |
| Valvular disease | 1,777 | 51 (2.9) |  | 1,299 | 29 (2.2) |  | 98 | 7 (7.1) | 0.014 |
| History of stroke/embolic event |  |  |  |  |  |  |  |  | 0.59 |
| Yes | 1,777 | 1,056 (59.4) |  | 1,299 | 803 (61.8) |  | 98 | 64 (65.3) |  |
| No |  | 95 (5.3) |  |  | 63 (4.8) |  |  | 4 (4.1) |  |
| Unknown |  | 626 (35.2) |  |  | 433 (33.3) |  |  | 31 (30.6) |  |
| Laboratory findings |  |  |  |  |  |  |  |  |  |
| Hemoglobin, g/dL | 1,732 | 13.9 (12.9–15.0) |  | 1,235 | 14.0 (12.9–14.8) |  | 94 | 13.9 (12.9–15.0) | 0.81 |
| Creatinine, mg/dL | 1,732 | 0.9 (0.7–1.0) |  | 1,242 | 0.8 (0.7–1.0) |  | 95 | 0.8 (0.7–1.0) | <0.001 |
| BNP, pg/mL | 1,333 | 75.4 (31.5–161) |  | 892 | 44.0 (19.8–96.8) |  | 68 | 50.6 (15.1–108.7) | <0.001 |
| NT-proBNP, pg/mL | 329 | 354 (98–887) |  | 296 | 219 (86–691) |  | 15 | 258 (80–969) | 0.087 |
| Echocardiography |  |  |  |  |  |  |  |  |  |
| LVEF, % | 1,701 | 63 (58-68) |  | 1,217 | 65 (60-69) |  | 88 | 64 (60-70) | <0.001 |
| LAD, mm | 1,681 | 40 (36-45) |  | 1,204 | 39 (34-42) |  | 85 | 40 (35-44) | <0.001 |
| Balloon technologies |  |  |  | 1,299 |  |  | 98 |  | <0.001 |
| Cryoballoon |  | – |  |  | 1,169 (90.0) |  |  | 76 (77.6) |  |
| Hot balloon |  | – |  |  | 59 (4.5) |  |  | 17 (17.3) |  |
| Laser balloon |  | – |  |  | 71 (5.5) |  |  | 5 (5.1) |  |
| Mapping technologies | 1,759 |  |  | 1,269 |  |  | 95 |  | <0.001 |
| Carto |  | 1,301 (74.0) |  |  | 173 (13.6) |  |  | 23 (24.2) |  |
| EnSite |  | 429 (24.4) |  |  | 693 (54.6) |  |  | 60 (63.2) |  |
| Rhythmia |  | 20 (1.1) |  |  | 3 (0.2) |  |  | 1 (1.1) |  |
| No mapping system |  | 9 (0.5) |  |  | 400 (31.5) |  |  | 11 (11.6) |  |
| Cardiac CT before ablation | 1,775 | 1,635 (92.1) |  | 1,299 | 1,265 (97.4) |  | 98 | 93 (94.9) | <0.001 |
| TTE before ablation | 1,775 | 897 (50.5) |  | 1,299 | 556 (42.8) |  | 98 | 41 (41.8) | <0.001 |
| Procedure time (min) | 1,628 | 180 (135-215) |  | 1,193 | 120 (90-150) |  | 86 | 148 (120-180) | <0.001 |
| Radiation time (min) | 1,524 | 23 (12-40) |  | 1,137 | 25 (17-39) |  | 81 | 32 (24-43) | <0.001 |
| Year | 1,777 |  |  | 1,299 |  |  | 98 |  | 0.22 |
| 2018 |  | 421 (23.7) |  |  | 346 (26.6) |  |  | 31 (32) |  |
| 2019 |  | 737 (41.5) |  |  | 523 (40.3) |  |  | 37 (38) |  |
| 2020 |  | 619 (34.8) |  |  | 430 (33.1) |  |  | 30 (31) |  |
| Data are presented as the number (%) or the median (IQR).  AF, atrial fibrillation; AAD, antiarrhythmic drug; BNP, B-type natriuretic peptide; CT, computed tomography; IHD, ischemic heart disease; LVEF, left ventricular ejection fraction; LAD, left atrium diameter; NT-proBNP, N-terminal-pro-BNP; TTE, transthoracic echocardiography | | | | | | | | | |

| Supplemental Table 2. Cox proportional hazard model for 1-year recurrence free | | | | | |
| --- | --- | --- | --- | --- | --- |
|  | Univariable analysis | |  | Multivariable analysis | |
|  | Hazard ratio (95% CI) | P-value |  | Hazard ratio (95% CI) | P-value |
| Radiofrequency ablation | 0.94 (0.56–1.58) | 0.82 |  | 0.77 (0.45–1.29) | 0.32 |
| Balloon ablation | ref |  |  | ref |  |
| Balloon ablation + touch-up | 0.71 (0.41–1.20) | 0.39 |  | 0.74 (0.44–1.27) | 0.28 |
| Multivariable analysis was adjusted with age, sex, body mass index, type of AF, AF symptoms, failed antiarrhythmic drugs, history of stroke or embolic event, and comorbidities.  CI, confidence interval | | | | | |
